# Optimization of microfluidic biosensor efficiency by means of fluid flow engineering

**DOI:** 10.1038/s41598-017-06204-0

**Published:** 2017-07-18

**Authors:** Marwa Selmi, Mohamed Hichem Gazzah, Hafedh Belmabrouk

**Affiliations:** 1 Laboratory of Electronics and Microelectronics, Faculty of Science of Monastir, University of Monastir, Environment Boulevard, Monastir, 5019 Tunisia; 2 Department of Radiological Sciences and Medical Imaging, College of Applied Medical Sciences, Majmaah University, 11952 AlMajmaah, Saudi Arabia; 3 Department of Physics, College of Science AlZulfi, Majmaah University, 11932 AlZulfi, Saudi Arabia

## Abstract

Binding reaction kinetics of analyte-ligand at the level of a sensitive membrane into a microchannel of a biosensor has been limited by the formation of the boundary diffusion layer. Therefore, the response time increases and affects the overall performance of a biosensor. In the present work, we develop an approach to engineer fluid streams into a complex configuration in order to improve the binding efficiency. We investigate numerically the flow deformations around a parallelepiped with square cross-section inside the microfluidic channel and exploit these deformations to simulate the analyte transport to the sensitive membrane and enhance both association and dissociation processes. The effect of several parameters on the binding reaction is provided such as: the obstacle location from the inlet of the microchannel, the average flow velocity, and the inlet analyte concentration. The optimal position of the obstacle is determined. An appropriate choice of the inlet flow velocity and inlet analyte concentration may reduce significantly the response time.

## Introduction

Microfluidics has gained tremendous attention over the last decade as an attractive research field^1–3^. Their development has been conditioned by the technological ability of adapting microfabrication techniques originally designed for electronic fluidic applications. These microfluidic devices provide many of the features that make bioassays advantageous, such as short analysis time and the ability to operate with small samples and high sensitivity^4^. Recently, there has been an increasing interest to integrate advanced biosensors into lab-on-a-chip systems by introducing microfluidics^5, 6^. Jang *et al*.^7^ presented in their work a simple method to fabricate microfluidic devices for potential applications as biosensors in micro-total-analysis-system (µ-TAS). Lee *et al*.^8^ have developed a chip based microfluidic device that has a multi-channel configuration to detect microarray immunoassay samples based on a surface plasmon resonance (SPR) detection system.

In clinical applications, quantitative analysis of proteins plays an important role in the diagnosis of diseases. For example, C-reactive protein (CRP) is a biomarker of inflammation whose concentration increases several hundred times in response to acute injury, infection, or tissue damage^9^. Integrated microfluidic system for rapid screening of CRP aptamers utilizing the systematic evolution of ligands by exponential enrichment has been developed by Huang *et al*.^10^.

The SPR sensor^11^, the quartz crystal microbalance (QCM) sensor^12, 13^, the impedance based sensor^14^ and the immunoassays are the most frequently used devices for the quantitative monitoring of biomolecules. Although the processes of detection are different, they all implicate the same kinetics of specific binding of analytes and immobilized ligands. More specifically, the system mixes a small concentration of a biological analyte, such as CRP, with the fluid in a microchannel. The binding efficiency on the reaction surface is usually large enough to bind practically all analyte molecules appearing there. Thus, the reaction is said to be transport limited and it usually causes the formation of a diffusion boundary layer^15, 16^. The development of the diffusion boundary layer increases the response time and limits the overall performance of the biosensor^17, 18^. In practice, a detection cycle can take many hours, which is a problem that must be solved.

Currently, there are ongoing scientific research efforts to develop several mechanical and physical strategies for replenishing the target antigen to the sensor surface and to enhance the mass transport in microfluidic networks. Hofmann *et al*.^19^ developed a three-dimensional microfluidic confinement device for efficient sample delivery to biosensor surfaces: application to immunoassays on planar optical waveguides. We expand the study of the flow confinement effect on the binding reaction in order to enhance efficient mass transport^20^. This technique has been studied in great details, for flow confinement, a sample flow is joined with a perpendicular makeup flow of water or sample medium. The makeup flow confines the sample into a thin layer above the sensing area. Therefore, the velocity flow increases to improve the binding rate. Sigurdson *et al*.^21^ suggested a technique to enhance microfluidic immuno- sensors by using AC electrokinetically-driven microscale fluid motion to enhance antigen motion towards immobilized ligands. The binding rate can be increased in the first few minutes by a factor of seven using a 6 Vrms applied potential. However, these observations are related only to the association phase of the binding reaction. Huang *et al*.^22^ performed a two dimensional full time scale finite element simulation on the binding reaction kinetics of two proteins (CRP) and immunoglobulin G (IgG). The electrothermal force results from the application of a non uniform ac electric field to the flow in the microchannel of the biosensor. It can generated a vortex field to stir the flow and reduce the thickness of the diffusion boundary layer. Consequently, the reaction rate is increased and the association and dissociation processes are accelerated. There is, however, a lack of thermal studies on the effect of the temperature rise on the chemical reaction. Zimmermann *et al*.^23^ modeled and optimized the reaction kinetics in the capillary-flow-driven microchannel immunoassays using finite difference method. Hu *et al*.^24^ designed a new poly(dimethylsiloxane) (PDMS) microfluidic chips for heterogeneous immunoassay using electrokinetic control. Hu *el al*.^25^ also presented in another work, a theoretical model that includes only the convection and diffusion of the bulk analyte flow and binding reactions at the solid surface. The used model shows that electrokinetically driven immunoassays have better reaction kinetics than pressure-driven ones, resulting from the plug-like velocity profile. Hart *et al*.^26^ studied the enhancement of heterogeneous immunoassays using AC electroosmosis (ACEO) through the use of finite element method and fluorescent immunoassays. They also indicated in their work that the heterogeneous immunoassay is enhanced by the use of combined ACEO and dielectrophoresis (DEP). Munir *et al*.^27^ developed a novel and simple strategy based on tagging analyte with MNPs towards the sensing zone by using magnetic field force. Amini *et al*.^28^ developed a hierarchical approach to engineer fluid streams into a board of complex configurations. They investigated the flow deformations around a set of cylinders within a microfluidic channel. In addition, utilizing this platform to guide liquid in microchannels is useful in a variety of applications. This technique presents a passive mechanism to stir the flow. It mixes the fluid quite effectively and does not increase temperature. It is more suitable for biomedical applications than active mechanism employing the electrothermal force. In our previous study, we have studied the combination of the effect of the flow confinement and the effect of the electrothermal force on the heterogeneous immunoassays as a promising approach to increase mass transport at the level of the sensitive surface of a biosensor^29^.

In this study, we develop a novel approach to engineer fluid streams in order to enhance the binding efficiency of immunoassay for a microfluidic biosensor. We will also investigate the flow deformations around a cylinder with square cross-section within a microfluidic channel and using fluid transformations to reduce the diffusion boundary layer. We will perform two dimensional simulations and study the effect of changing the geometrical locations of the obstacle on the biosensor performance. In addition, we examine some important factors such as the average inlet flow velocity, and the initial analyte concentration that control the immunoreaction in the microfluidic biosensor. The sample of CRP pairs in a biosensor immunoassay will be used to predict the response time of the biosensor as a function of the previous parameters.

## Theoretical Formulation

### Balance equations

#### Fluid flow

In the microfluidic channel, the flow velocity field is described by the Navier-Stokes equations. The fluid is assumed to be Newtonian and incompressible. In the 2D Cartesian coordinates and for a steady flow, the conservation and Navier-Stokes equations can be written as:

1 $$\frac{\partial u}{\partial x}+\frac{\partial v}{\partial y}=0$$

2 $$\{\begin{array}{c}\rho (u\frac{\partial u}{\partial x}+v\frac{\partial u}{\partial y})=-\frac{\partial P}{\partial x}+\mu (\frac{{\partial }^{2}u}{\partial {x}^{2}}+\frac{{\partial }^{2}u}{\partial {y}^{2}})\\ \rho (u\frac{\partial v}{\partial x}+v\frac{\partial v}{\partial y})=-\frac{\partial P}{\partial y}+\mu (\frac{{\partial }^{2}v}{\partial {x}^{2}}+\frac{{\partial }^{2}v}{\partial {y}^{2}})\end{array}$$

where *P* is the pressure, (*u*, *v*) the Cartesian components of the velocity vector. The fluid properties such as the dynamic viscosity and the density are assumed to be constant. The dynamic viscosity is *μ* = 10^−3^ *Pa*.*s*, and the fluid density is *ρ* = 1000 kg/m^3^.

#### Analyte transport model

A biological analyte is introduced with the fluid in a microchannel containing a sensitive surface. The analyte may be C-reactive protein (CRP) and its concentration is very small so that the density and viscosity of the fluid are not changed. In this study, the initial analyte [A_0_] varies in the range 6.4 × 10^−6^ mol/m^3^ to 22 × 10^−6^ mol/m^3^. However, the initial surface concentration of the ligand is constant [B_0_] = 1.8 × 10^−8^ mol/m^2^. The molecule weight of human CRP is 115 kDa. It consists of five identical noncovalently bonded monomer subunits linked in the form of a cyclic pentamer^30^. The CRP is known as a marker for infections and inflammatory processes in human blood serum. CRP is usually present in human serum with a concentration <1 µg/ml. Nevertheless, CRP concentration level can increase up to tens or even hundreds times when the inflammation occurs.

The analyte is then carried to the sensitive surface due to diffusion and convection. We denote by [A](x, y, t) the concentration of the analyte inside the flow and [A]_surface_ the analyte concentration at the sensitive surface. Obviously, [A]_surface_ is involved in binding reaction and the variations of [A](x, y, t) are due to the transport phenomena.

The spatial and temporal variations of the analyte inside the microfluidic channel are described by the convection-diffusion equation:

3 $$\frac{\partial [{\rm{A}}]}{\partial {\rm{t}}}+{\rm{u}}\frac{\partial [{\rm{A}}]}{\partial {\rm{x}}}+{\rm{v}}\frac{\partial [{\rm{A}}]}{\partial {\rm{y}}}={\rm{D}}(\frac{{\partial }^{2}[{\rm{A}}]}{\partial {{\rm{x}}}^{2}}+\frac{{\partial }^{2}[{\rm{A}}]}{\partial {{\rm{y}}}^{2}})+{\rm{G}}$$

where the [A] is the analyte concentration in bulk, D is the analyte diffusion coefficient (D = 2.175 × 10^−11^ m^2^/s) and G denotes the reaction rate. Here G equals to zero because no reactions take place in the fluid bulk.

#### The binding reaction

The analyte portion diffused towards the sensitive membrane reacts with the antibody ligands immobilized on the reaction surface. The chemical reaction process is as follows:

4 $${A}_{surf}+B{\rightleftharpoons }_{{k}_{off}}^{{k}_{on}}AB$$

The chemical reaction can be observed as being an adsorption process since it takes place at the binding surface. Many models have been used to describe the adsorption phenomena. Some of them are empirical models whereas some others are based on statistical physics^31, 32^. In this work, we will use the first order Langmuir adsorption model^15, 22, 33^. This model is often used to study the dynamics of molecular reactions in biochips and SPR biosensors^23, 34^.

The complex concentration [AB] produced during the reaction increases as a function of time until saturation. The binding rate depends on the analyte concentration on the surface [*A*_*surf*_], the concentration of free antibodies [B] and the association rate constant *k*_*on*_. Similarly, the dissociation reaction rate depends on the concentration of bonded ligands [AB] and on the dissociation rate constant *k*_*off*_. However, the concentration of free antibodies is equal to the difference between the total concentration of free antibodies [B_0_] and the concentration of bonded ligands [B] = [B_0_] − [AB].

5 $$\frac{d[AB]}{dt}={k}_{on}[{A}_{surf}](t)\{[{B}_{0}]-[AB](t)\}-{k}_{off}[AB](t)$$

In addition, we assume that the antibodies do not diffuse on the surface and that there is no leakage of the molecules at the edges of the surface. The association and dissociation constants, i.e. *k*_*on*_ and *k*_*off*_ for CRP-anti-CRP binding interactions are 10^7^ M^−1^s^−1^ and 2.6 × 10^−2^ s^−1^, respectively.

### Microfluidic immunoassays geometry

**Figure 1:**
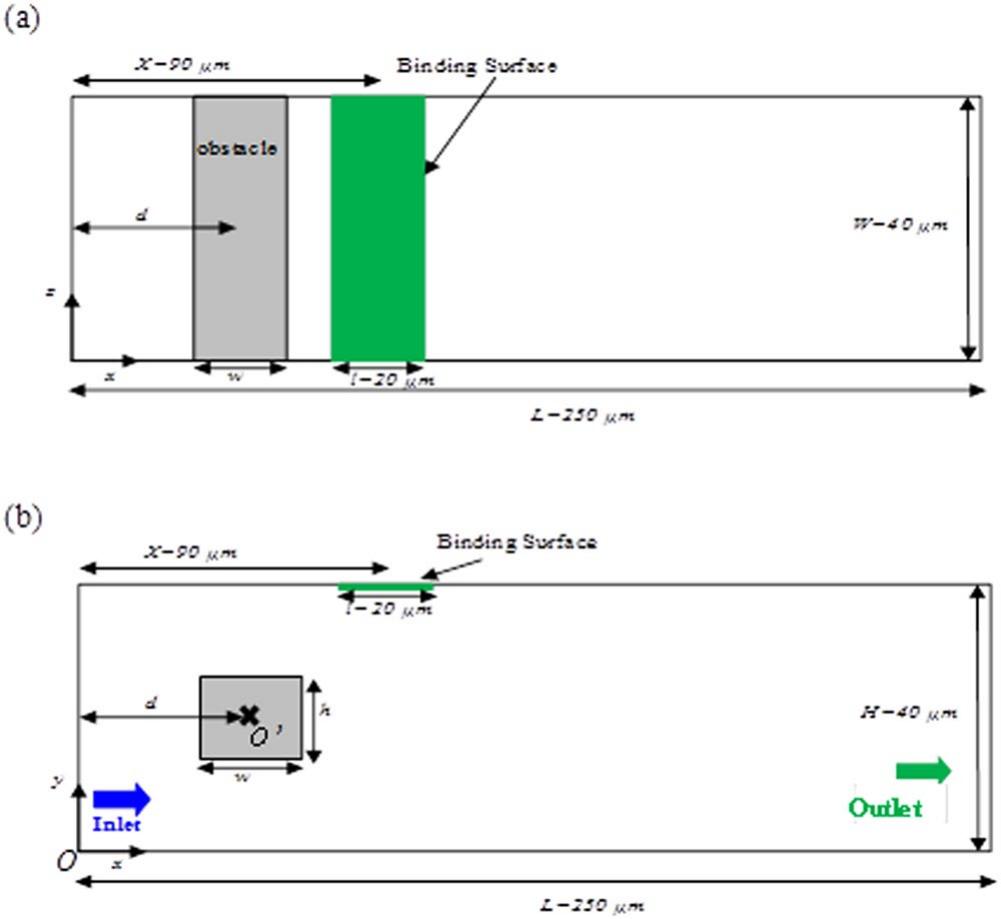
Microchannel containing a sensitive membrane and an obstacle with square cross-section, (**a**) top view, (**b**) front view.

We examine transport dynamics using a model geometry of a surface plasmon resonance sensor (SPR). The originality of the present work consists on the insertion of a cylindrical obstacle with square cross-section inside the channel (Fig. 1).

The dimensions of the biosensor and the microchannel are marked in the same figure. The length scales of the obstacle are *w* = *h* = *20* µm. *O’*(*x* = *d*, *y* = *H/2*) is the center coordinates of the square, *d* is the lengthwise position of center from the inlet and H is the channel height. The binding surface (*B*-*S*), located at the top of the microchannel, has a length of *l* = *20* µm.

### Boundary and initial conditions

In the matter of the Navier-Stokes equations, the following boundary conditions have been used.

i. At the inlet of the microchannel, the fluid flows in the lengthwise direction (x), with a parabolic velocity profile along the direction (y):
  $$u(0,y)=4{v}_{ave}\frac{y}{H}(1-\frac{y}{H})$$
  $$v(0,y)=0$$
ii. A non-slip boundary condition at the lower solid boundary is applied. The velocity is therefore set to zero at this wall:
  $$u(x,0)=v(x,0)=0$$
iii. The same boundary condition is applied at the upper wall:
  $$u(x,H)=v(x,H)=0$$
iv. At the outflow boundary, the gradient of the axial velocity in the axial direction and the radial velocity were set to zero:

$$\frac{\partial u}{\partial x}(L,y)=0$$

In the matter of the mass-transport equation (eq. 3), the boundary conditions are:

i. The inlet condition which consists of a uniform concentration: [*A*](0, *y*, *t*) = [*A*_0_];
ii. At the lower wall.
  This wall is assumed to be impermeable and does not interact with the analyte:
  $$\frac{\partial [A]}{\partial y}(t,\,x,0)=0$$
iii. The upper wall contains the binding surface (*y* = *H*, $X-\frac{l}{2}\le x\le X+\frac{l}{2}$) and the remaining wall.
  At the binding surface, the diffusion flux is balanced against the reaction rate. This boundary condition reads:
  $${\rm{D}}{(\frac{\partial [{\rm{A}}]}{\partial {\rm{y}}})}_{{\rm{surface}}}=-{{\rm{k}}}_{{\rm{on}}}{[{\rm{A}}]}_{{\rm{surface}}}({[{\rm{B}}]}_{0}-[{\rm{AB}}])-{{\rm{k}}}_{{\rm{off}}}[{\rm{AB}}]$$
  The remaining part of the upper wall is assumed to be impermeable and does not interact with the analyte. Therefore, we have:
  $$\frac{\partial [{\rm{A}}]}{\partial {\rm{y}}}({\rm{t}},{\rm{x}},{\rm{H}})=0\,(0\le {\rm{x}}\le {\rm{X}}-{\rm{l}}/2\,{\rm{or}}\,{\rm{X}}+{\rm{l}}/2\le {\rm{x}}\le {\rm{L}})$$
iv. At the outlet, the profile of the analyte concentration is assumed to be fully established. We adopted the boundary condition:

$$\frac{\partial [{\rm{A}}]}{\partial {\rm{x}}}({\rm{t}},{\rm{L}},{\rm{y}})=0$$

The initial conditions for both the concentrations of the analyte in the bulk, [*A*]_*bulk*_(0, *x*, *y*), and the concentrations of the analyte–ligands complex on the reaction surface, [*AB*](0, *x*, *y*), are all zero. The initial surface concentration [B_0_] is assumed as 1.8 × 10^−8^ mol/m^2^.

### Numerical method and model validation

**Figure 2:**
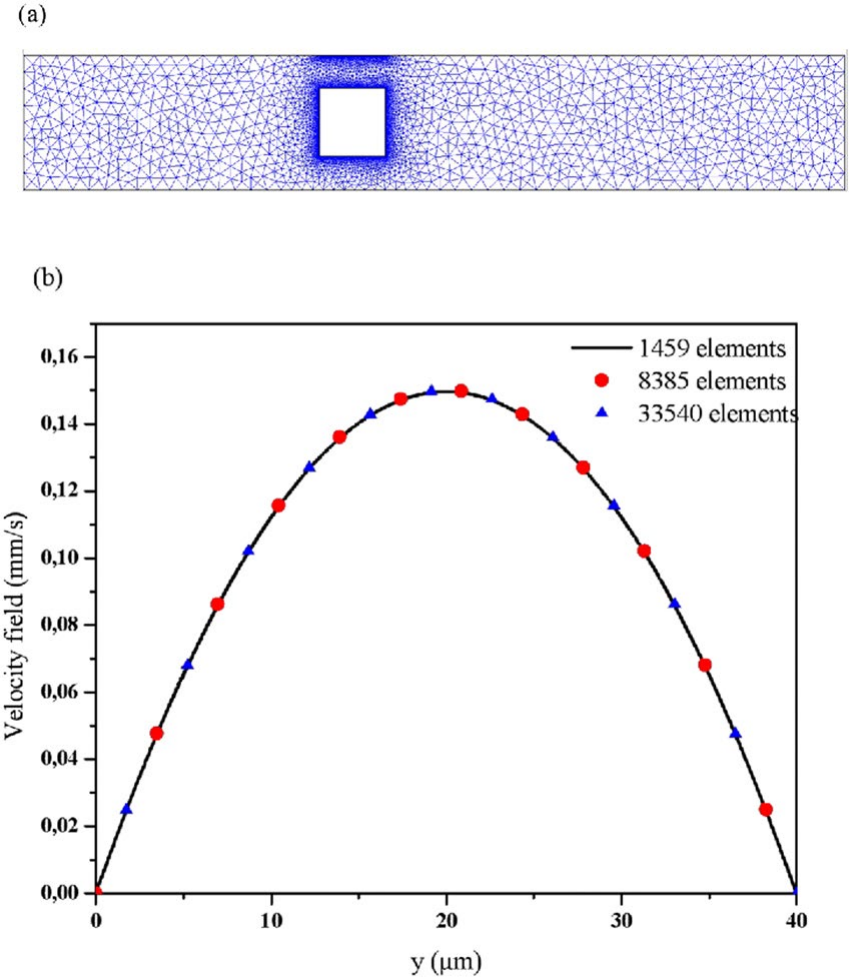
(**a**) 2D unstructured mesh with triangular elements, (**b**) velocity field at the outlet of the microchannel for several meshes.

The system of the transport equations coupled with the first Langmuir adsorption model is solved using the finite element method (FEM) with the Galerkin method^35^. To find the numerical solution to these equations, a computer code was developed^36^. Firstly, the 2D domain is divided in triangular elements as presented in Fig. 2a. The regions nearby the obstacle and the reaction surface are refined with a better mesh quality. Secondly, all the variables (i.e. the velocity components *u*(*x*, *y*), *v*(*x*, *y*) are approximated using second order interpolation and first order for the pressure in each element whereas the analyte concentration [*A*](*t*, *x*, *y*) is approximated using quadratic interpolation in each element^37^.

To ensure that the convergence has been reached and the computed results are independent of the mesh size, Fig. 2b shows the velocity field at the outlet the microchannel for several mesh grids, namely, 1454, 8385, and 33540 elements. The results presented hereafter are obtained with a total number 8385 elements and a refined mesh grid near the sensitive surface.

For the calculation, we solve firstly, the Navier-Stokes equations to obtain the velocity field. Then, we solve the analyte transport equation coupled with the complex concentration equation. These two equations are time-dependant. The total concentration accumulated at the capture area can be found by integrating local concentration over the whole reaction surface.

### Model validation against experimental data

**Figure 3:**
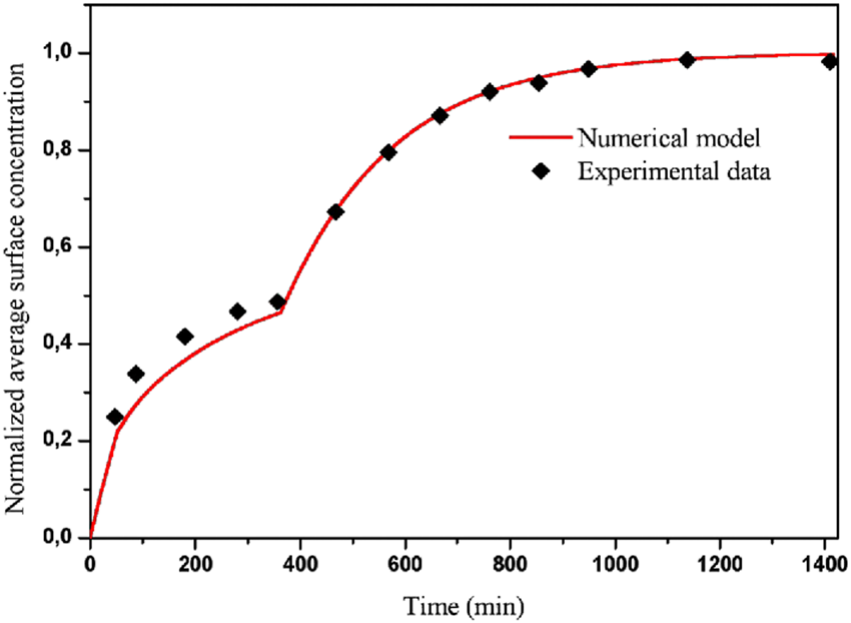
Comparison between numerical and experimental temporal evolutions of the normalized average surface concentration in a particular configuration.

The validation of the numerical model in this work for the mass transport inside the microchannel is performed on the basis of the experimental data for 29 basis pairs of DNA strands from the literature^38^. In the experiment, the hybridization kinetics of the DNA strands is controlled by the fluorescence microscopy, and the flow is turned on for 50 min, then turned off for 310 min and again turned on for the rest. The total interval of the experiment is 1400 min. When the flow is turned off, only the diffusion mechanism takes place. However, when the flow is turned on, there is a competition between the diffusion and convection mechanisms. Figure 3 shows the temporal evolution of the normalized average surface concentration.

Our numerical results are compared with the experimental data available in the literature^38^. The numerical result presents a good agreement with the experimental data. This proves that the model used to describe the kinetic reaction is valid. It is worthy to notice that other works validate the first order Langmuir model for other complexes^21, 25, 33, 39^. We deduce that this model can describe suitably the mechanisms inherent to the association and dissociation phases of the binding reaction. This proves that the surface concentration of the complex AB is computed accurately. The characteristic of the binding reaction is in quantitative agreement with the theory works. The results obtained with the present model have also the same behavior as the experimental and numerical results presented by Han and Park^40^.

## Results and Discussion

Rapid detection time of biomolecules is a major challenge for microfluidic-based biosensor. Especially, detection time for heterogeneous immunoassays is long because the analyte takes a long time to transport by convection and diffusion to the sensing zone to bind with the surface-bound receptors. The limitation of mass transport presents a critical barrier which restricts the performances of the devices. It should be overcome for many biosensor applications^15, 17, 41, 42^. For this purpose, we investigated numerically the flow deformations around a parallelepiped with square cross-section inside the microfluidic channel on the binding reaction.

**Figure 4:**
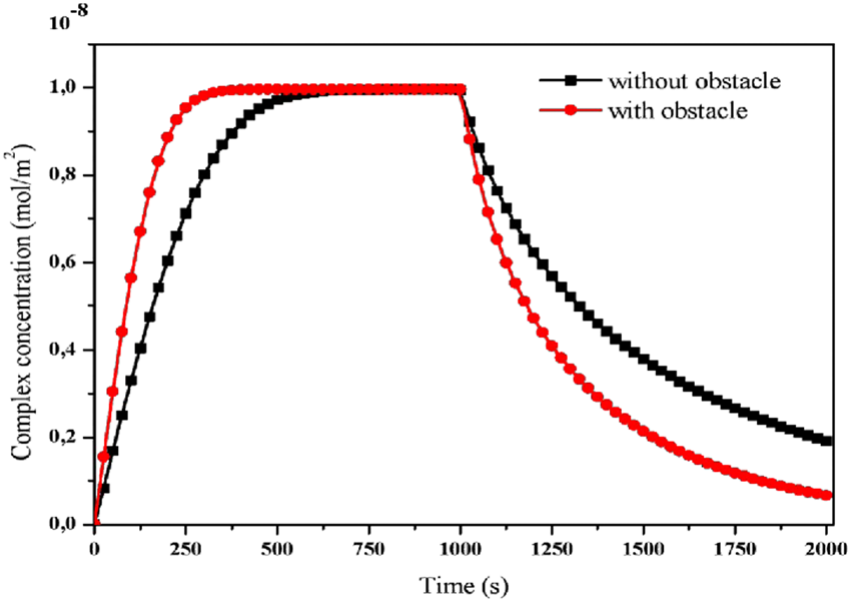
Temporal evolution of the complex concentration without and with obstacle.

Figure 4 shows the evolution of the average complex concentration of CRP-anti-CRP with and without insertion of the obstacle inside the microchannel. The initial analyte concentration [A_0_] = 6.4 10^−6^ mol/m^3^ and *v*_*ave*_ = 100 µm/s. The concentration increases steeply over a short time and then it reaches a saturation value. The time (t = 1000 s) of interrupting the injection of the analyte is set manually. We defined the equilibrium binding time T_R_ as the response time of biosensor the time for which the average concentration equals 95% of its saturation value. For a channel without obstacle, we obtained T_R_ = 440 s whereas T_R_ = 242 s when the obstacle is included. We observed also when the obstacle is involved, the evolution of the complex concentration declines rapidly during the dissociation phase.

**Figure 5:**
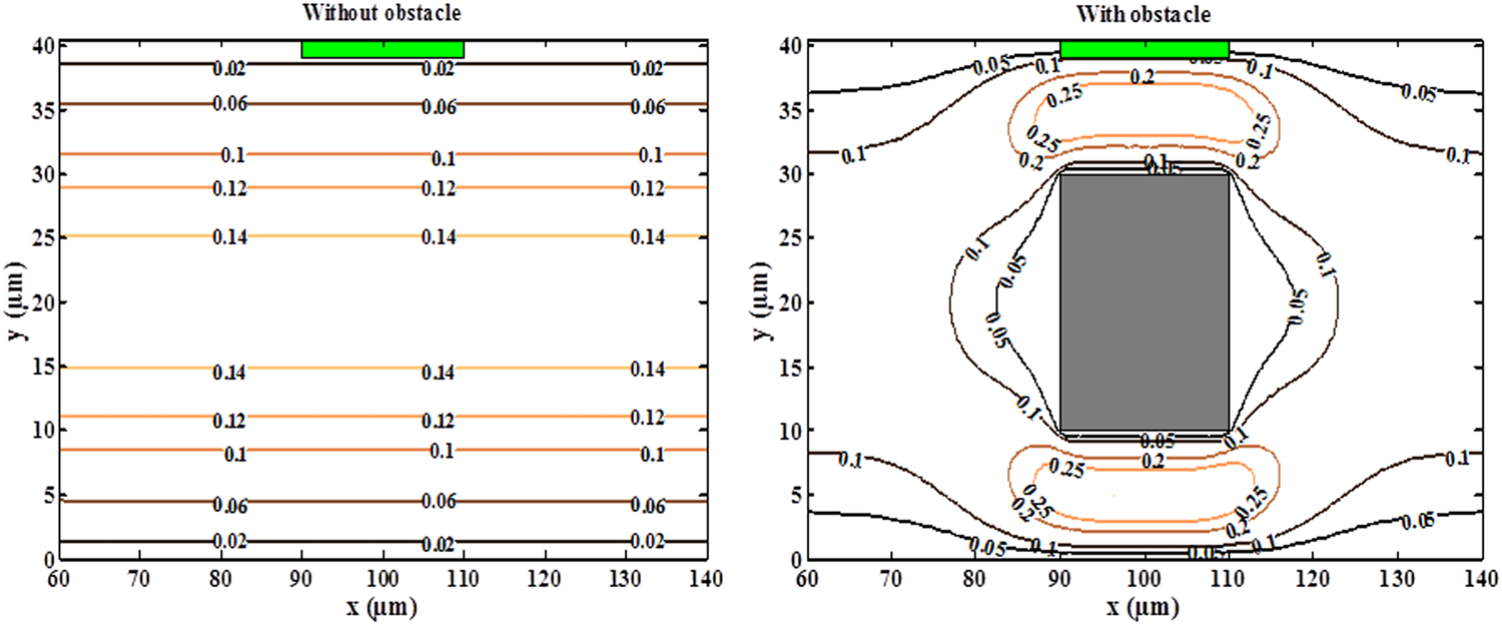
Velocity contours for the flow without and with the obstacle.

The numerical result proves the usefulness of the introduction of an obstacle to reduce T_R_ and improve the biosensor response. The effect of the obstacle may be explained by the deformation of the streamlines and the modification of the fluid topology as shown in Fig. 5. In fact, the obstacle obstructs a part of the cross-section and leads to an increase of the velocity in the vicinity of the sensitive membrane. For instance, in the fourth case, the velocity reaches the value 0.25 mm/s. The perturbation around the obstacle limits the formation of the diffusion boundary layer of the analyte concentration behind the sensitive surface. These results agree with those obtained by Amini *et al*.^28^. Indeed, the obstacle restrains the cross-section of the channel and leads to an increase of the velocity near the sensing area of the biosensor. Our numerical results present a good agreement with experimental results performed by Hofmann *et al*.^19^ and theoretical results obtained by Hu *et al*.^25^. Indeed, they found that the equilibrium binding time could be reduced by increasing the flow rate.

### Influence of the obstacle position on the binding reaction

**Figure 6:**
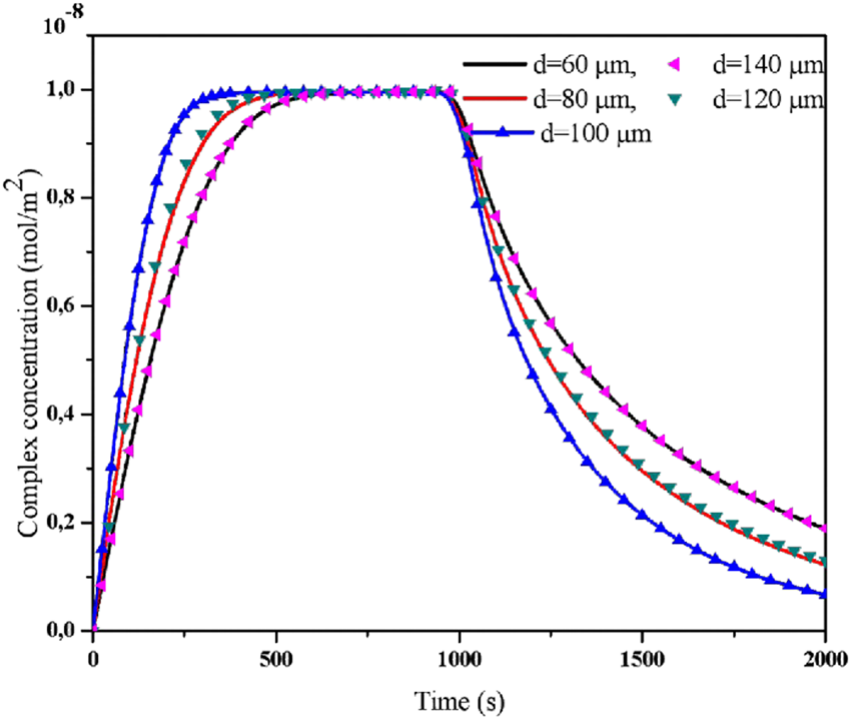
Temporal evolution of the complex concentration for five obstacle positions.

To determine the optimal position of the obstacle inside the microchannel, Fig. 6 depicts the temporal evolution of the complex concentration for five positions of the obstacle, namely d = 60, 80, 100, 120 and 140 µm. when the obstacle approaches the binding surface, the response time decreases and reaches its smallest value when d = 100 µm. At this value corresponds the center of the obstacle is situated in the front of the center of the binding surface. Then, when the obstacle move away from the optimal position (d = 100 µm) the response time increases. We note also, there is some symmetry in the positions of the obstacle. Indeed, the curves related to d = 60 µm and d = 80 µm are respectively identical to the curves related to d = 140 µm and d = 120 µm.

The optimal position giving the smallest T_R_ corresponds to d = 100 µm. The enhancement of the binding reaction may be explained by the deformation of the streamlines and the modification of the fluid velocity field which leads to increase the velocity in the vicinity of the sensitive membrane. This deformation is more advantageous when the obstacle is located in front of the center of the sensitive membrane.

### Influence of the inlet flow velocity on the binding reaction

**Figure 7:**
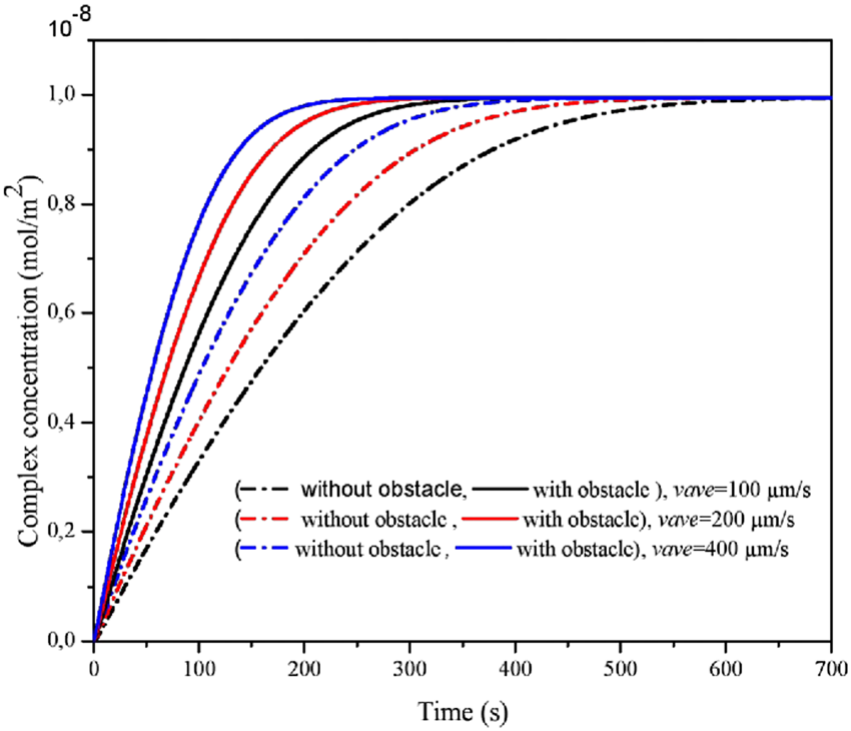
Temporal evolution of the complex concentration for three values of the inlet velocity and with/without obstacle.

Raising flow velocity is an effective means to reduce the thickness of the diffusion boundary layer^33^. Indeed, Fig. 7 displays the average complex concentration of the CRP-anti-CRP without obstacle for three values of the inlet flow velocity and for d = 100 µm. These curves are plotted only during the association phase. The increase of the inlet velocity induces the decrease of the response time T_R_. Figure 7 shows also the temporal evolution of the complex concentration for the same values of the inlet velocity but using the obstacle. It is clear that the presence of the obstacle has an important effect on the binding reaction. The evolution of complex concentration rises with raising the flow velocity as already mentioned and the presence of the obstacle leads to a further decrease of the response time. It is interesting to observe that a “squeezing” effect has caused the velocity of the flow between the boundary and the obstacle to be mostly increased.

**Figure 8:**
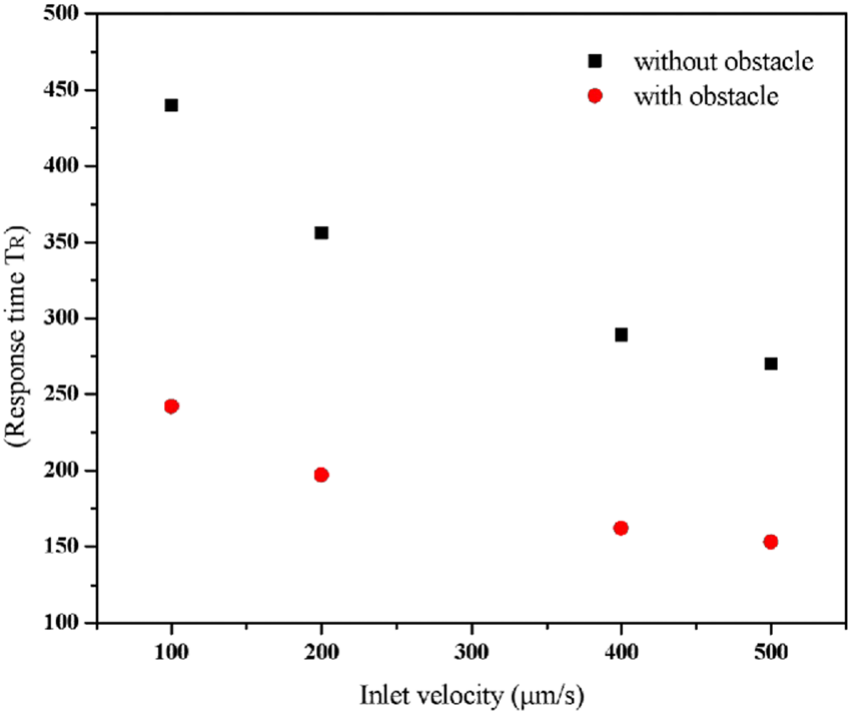
Effect of the presence of the obstacle on the response time.

To apprehend more quantitatively the effect of the presence of the obstacle on the binding reaction, Fig. 8 presents the response time for several values of the inlet velocity and with or without using the obstacle. This figure confirms that the increase of the inlet velocity leads to reduce of the response time in the both configurations. For the same inlet velocity, Fig. 8 shows clearly that the presence of the obstacle reduces by a factor 1.8 the response time. This figure indicates also that to obtain the same response time T_R_, the inlet velocity in the case of using the obstacle reduced by a factor about 10 compared with the case that the obstacle is not included. We conclude that the insertion of an obstacle inside a microchannel is an effective means to reduce the response time instead of increasing the inlet velocity.

**Figure 9:**
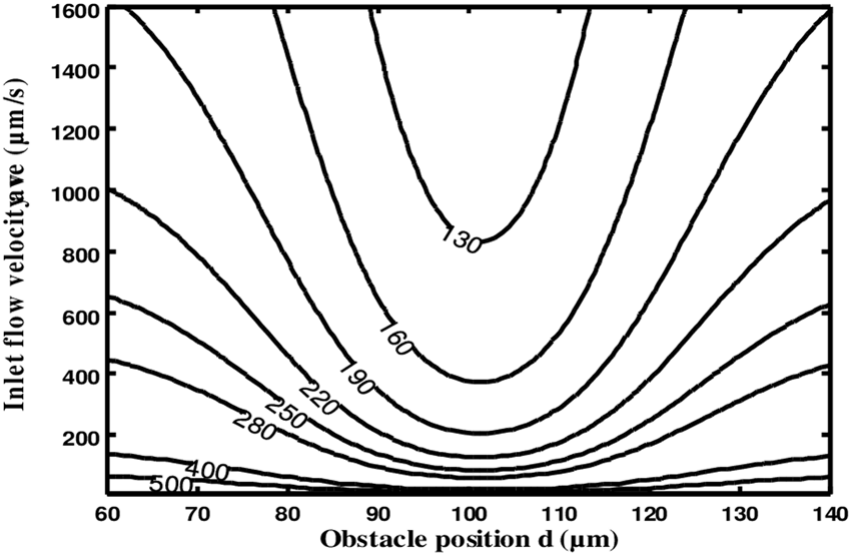
Contour lines of the equilibrium binding time T_R_ as a function of the obstacle position *d* and the inlet flow velocity *v*_*ave*_.

Figure 9 illustrates the contour lines of the response time versus the obstacle position d and the initial inlet flow velocity *v*_*ave*_. For each case, the analyte concentration at the inlet is constant (=6.4 nM). This figure shows simultaneously the effect of two parameters on the equilibrium binding time. It is then possible to deduce the best way to reduce T_R_. It is clear that the reduction of the response time is proportional to an increase of the flow velocity. It is noted also that the optimal position corresponds to d = 100 µm.

### Influence of the inlet analyte concentration on the binding reaction

**Figure 10:**
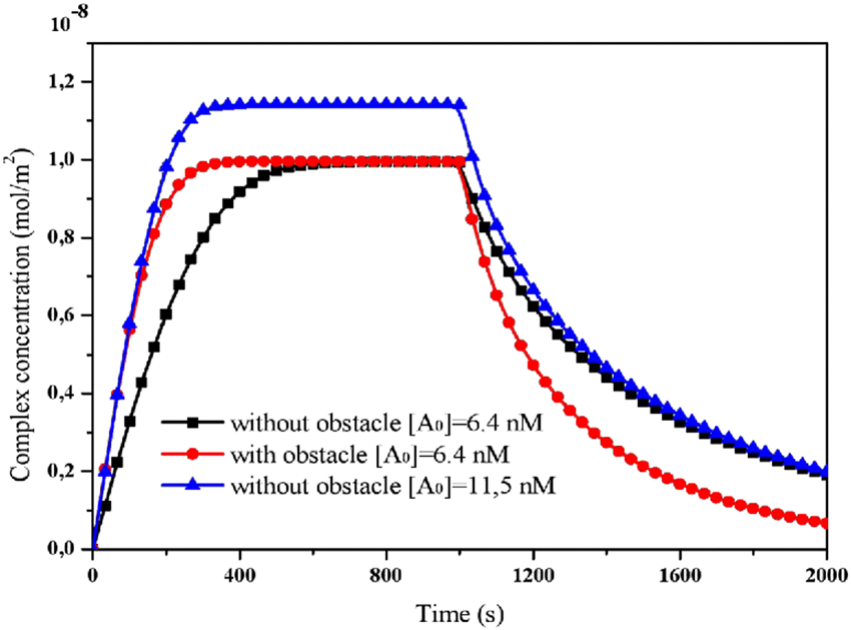
Temporal profiles of the normalized complex concentration for several values of the inlet analyte concentrations.

The enhancement of the mass transport comes at the increase of the inlet analyte concentration^25^. Indeed, Fig. 10 shows the evolution of the complex concentration with and without the obstacle for two different values of the inlet analyte concentration. The inlet flow velocity is set at 100 µm/s, and the obstacle is located at the optimal position. We observe that the consumption of the analyte [A] in the association phase is faster in the presence of the obstacle than when it is removed. It is clear that the rise of the inlet analyte concentration leads to an important increase of the initial curve slope and thus to a decrease of the equilibrium binding time. There is also further enhancement of the T_R_ with minimized consumption of the analyte concentration by using the obstacle inside the microchannel. Therefore, to obtain a given value of the response time it is more efficient to involve the obstacle than to increase the inlet analyte concentration.

**Figure 11:**
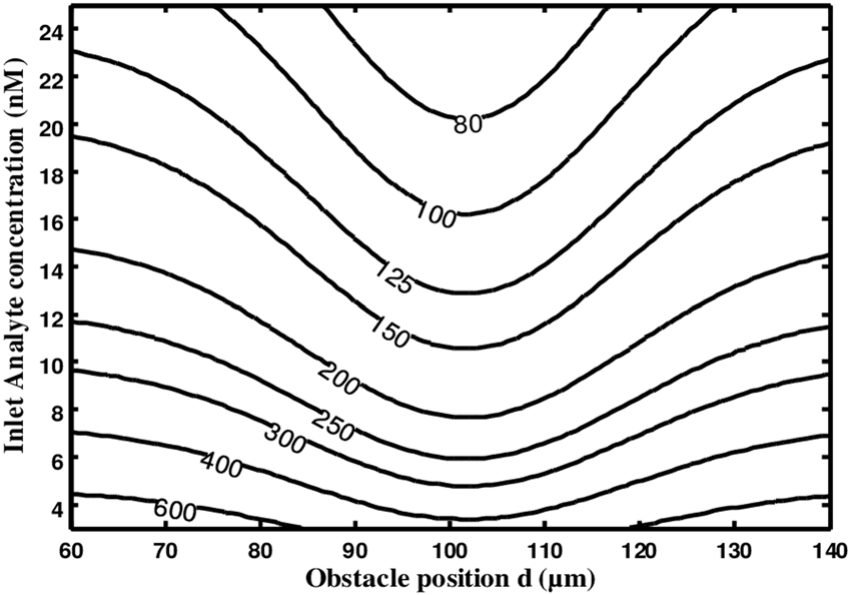
Contour lines of the equilibrium binding time T_R_ as a function of the obstacle position *d* and the inlet analyte concentration.

Figure 11 shows the contour lines of the response time T_R_ versus the obstacle position d and the inlet analyte concentration. The inlet flow velocity is fixed at 100 µm/s. The smallest equilibrium binding time is reached when the obstacle position is placed near the reaction surface, i.e. at the position (100, 20) µm which is in fact the optimal position regardless of the value of the inlet analyte concentration.

By an appropriate choice of the obstacle position, inlet flow velocity and the inlet analyte concentration, the response time can be significantly reduced as revealed in Figs 7 and 10. Thereby, the insertion of the obstacle is truly an effective way to enhance the binding reaction.

## Conclusion

This paper presents a numerical simulation of the effect of the flow deformations around an obstacle on the binding reaction inside a microchannel of a biosensor based on heterogeneous immunoassays. 2D numerical simulation is performed using the finite element method to study the effects of obstacle position, average inlet flow velocity, and initial analyte concentration on the response time. The results show that the obstacle yields better binding efficiency of a biosensor when it’s located in front of the center of the sensitive membrane. An appropriate choice of the inlet flow velocity and inlet analyte concentration may reduce significantly the response time.

In the future, we intend to expand the investigation in three dimensional simulations and we intend to examine in more details the bio-chemical models related to the adsorption.
